# Supplementary material for: HIVprotI: an integrated web based platform for prediction and design of HIV proteins inhibitors
Source: J Cheminform. 2018 Mar 9;10:12. doi: 10.1186/s13321-018-0266-y (PMC5845081; doi:10.1186/s13321-018-0266-y)
Supplement: Supplementary file 2 — Additional file 2. Source code of HIVProtI web server. [file 13321_2018_266_MOESM2_ESM.zip › HIVprotI_Source-code/hivprotI/weka-3-6-6/doc/weka/classifiers/bayes/net/estimate/BayesNetEstimator.html]

BayesNetEstimator


---


|  |  |  |
| --- | --- | --- |
| **Overview** \| **Package** \| **Class** \| **Tree** \| **Deprecated** \| **Index** \| **Help** \| **Weka's home** | |  |
| PREV CLASS   **NEXT CLASS** | **FRAMES**    **NO FRAMES**     **All Classes** |  |
| SUMMARY: NESTED \| FIELD \| CONSTR \| METHOD | DETAIL: FIELD \| CONSTR \| METHOD |  |


---


## weka.classifiers.bayes.net.estimate Class BayesNetEstimator

```
java.lang.Object
  weka.classifiers.bayes.net.estimate.BayesNetEstimator
```

**All Implemented Interfaces:**: java.io.Serializable, OptionHandler, RevisionHandler

**Direct Known Subclasses:**: MultiNomialBMAEstimator, SimpleEstimator

---

``` public class BayesNetEstimator extends java.lang.Object implements OptionHandler, java.io.Serializable, RevisionHandler ```

BayesNetEstimator is the base class for estimating the conditional probability tables of a Bayes network once the structure has been learned.


Valid options are:

```
 -A <alpha>
  Initial count (alpha)
```

**Version:**
:   $Revision: 1.4 $

**Author:**
:   Remco Bouckaert (rrb@xm.co.nz)

**See Also:**: Serialized Form

---

**Constructor Summary**

`BayesNetEstimator()`


| **Method Summary** | |
| --- | --- |
| `java.lang.String` | `alphaTipText()` |
| `double[]` | `distributionForInstance(BayesNet bayesNet, Instance instance)`             Calculates the class membership probabilities for the given test instance. |
| `void` | `estimateCPTs(BayesNet bayesNet)`             estimateCPTs estimates the conditional probability tables for the Bayes Net using the network structure. |
| `double` | `getAlpha()`             Get prior used in probability table estimation |
| `java.lang.String[]` | `getOptions()`             Gets the current settings of the classifier. |
| `java.lang.String` | `getRevision()`             Returns the revision string. |
| `java.lang.String` | `globalInfo()`             This will return a string describing the class. |
| `void` | `initCPTs(BayesNet bayesNet)`             initCPTs reserves space for CPTs and set all counts to zero |
| `java.util.Enumeration` | `listOptions()`             Returns an enumeration describing the available options |
| `void` | `setAlpha(double fAlpha)`             Set prior used in probability table estimation |
| `void` | `setOptions(java.lang.String[] options)`             Parses a given list of options. |
| `void` | `updateClassifier(BayesNet bayesNet, Instance instance)`             Updates the classifier with the given instance. |

**Methods inherited from class java.lang.Object**

`equals, getClass, hashCode, notify, notifyAll, toString, wait, wait, wait`

**Constructor Detail**

### BayesNetEstimator

```
public BayesNetEstimator()
```


**Method Detail**

### estimateCPTs

```
public void estimateCPTs(BayesNet bayesNet)
                  throws java.lang.Exception
```

:   estimateCPTs estimates the conditional probability tables for the Bayes
    Net using the network structure.

    :   **Parameters:**: `bayesNet` - the bayes net to use **Throws:**: `java.lang.Exception` - always throws an exception, since subclass needs to be used

---


### updateClassifier

```
public void updateClassifier(BayesNet bayesNet,
                             Instance instance)
                      throws java.lang.Exception
```

:   Updates the classifier with the given instance.

    :   **Parameters:**: `bayesNet` - the bayes net to use: `instance` - the new training instance to include in the model **Throws:**: `java.lang.Exception` - always throws an exception, since subclass needs to be used

---


### distributionForInstance

```
public double[] distributionForInstance(BayesNet bayesNet,
                                        Instance instance)
                                 throws java.lang.Exception
```

:   Calculates the class membership probabilities for the given test
    instance.

    :   **Parameters:**: `bayesNet` - the bayes net to use: `instance` - the instance to be classified **Returns:**: predicted class probability distribution **Throws:**: `java.lang.Exception` - always throws an exception, since subclass needs to be used

---


### initCPTs

```
public void initCPTs(BayesNet bayesNet)
              throws java.lang.Exception
```

:   initCPTs reserves space for CPTs and set all counts to zero

    :   **Parameters:**: `bayesNet` - the bayes net to use **Throws:**: `java.lang.Exception` - always throws an exception, since subclass needs to be used

---


### listOptions

```
public java.util.Enumeration listOptions()
```

:   Returns an enumeration describing the available options

    :   **Specified by:**: `listOptions` in interface `OptionHandler`
    :   **Returns:**: an enumeration of all the available options

---


### setOptions

```
public void setOptions(java.lang.String[] options)
                throws java.lang.Exception
```

:   Parses a given list of options.


    Valid options are:

    ```
     -A <alpha>
      Initial count (alpha)
    ```

    :   **Specified by:**: `setOptions` in interface `OptionHandler`
    :   **Parameters:**: `options` - the list of options as an array of strings **Throws:**: `java.lang.Exception` - if an option is not supported

---


### getOptions

```
public java.lang.String[] getOptions()
```

:   Gets the current settings of the classifier.

    :   **Specified by:**: `getOptions` in interface `OptionHandler`
    :   **Returns:**: an array of strings suitable for passing to setOptions

---


### setAlpha

```
public void setAlpha(double fAlpha)
```

:   Set prior used in probability table estimation

    :   **Parameters:**: `fAlpha` - representing prior

---


### getAlpha

```
public double getAlpha()
```

:   Get prior used in probability table estimation

    :   **Returns:**: prior

---


### alphaTipText

```
public java.lang.String alphaTipText()
```

:   **Returns:**: a string to describe the Alpha option.

---


### globalInfo

```
public java.lang.String globalInfo()
```

:   This will return a string describing the class.

    :   **Returns:**: The string.

---


### getRevision

```
public java.lang.String getRevision()
```

:   Returns the revision string.

    :   **Specified by:**: `getRevision` in interface `RevisionHandler`
    :   **Returns:**: the revision


---


|  |  |  |
| --- | --- | --- |
| **Overview** \| **Package** \| **Class** \| **Tree** \| **Deprecated** \| **Index** \| **Help** \| **Weka's home** | |  |
| PREV CLASS   **NEXT CLASS** | **FRAMES**    **NO FRAMES**     **All Classes** |  |
| SUMMARY: NESTED \| FIELD \| CONSTR \| METHOD | DETAIL: FIELD \| CONSTR \| METHOD |  |


---
